# Superficial Parasternal Intercostal Plane Blocks (SPIB) With Buprenorphine, Magnesium, and Bupivacaine for Management of Pain in Coronary Artery Bypass Grafting

**DOI:** 10.7759/cureus.30964

**Published:** 2022-11-01

**Authors:** Sandeep Krishnan, Ronak Desai, Peter Paik, Adam Cassella, Jon Lucaj, Farhad Ghoddoussi, Joffer Hakim, Charles Schwartz, Timothy Leicht, Kinjal Patel

**Affiliations:** 1 Anesthesiology, Wayne State University School of Medicine, Detroit, USA; 2 Anesthesiology, Cooper University Hospital, Camden, USA; 3 Anesthesiology, Cooper Medical School of Rowan University, Camden, USA; 4 Anesthesiology, Private Practitioner, Mansfield, USA; 5 Anesthesiology, Children's Hospital of Michigan, Detroit, USA; 6 Anesthesiology, Cedars-Sinai Medical Center, Los Angeles, USA; 7 Cardiothoracic Surgery, St. Joseph Mercy Oakland Hospital, Pontiac, USA

**Keywords:** post-operative pain management, post-operative opioid consumption, buprenorphine, magnesium, coronary artery bypass grafting, superficial parasternal intercostal block

## Abstract

Introduction

Management of post-operative pain after cardiac surgery continues to be a challenge; inadequate management of pain can lead to increased morbidity, impaired physical function with potential delay in recovery, increased perioperative and chronic opioid consumption, increased cost of care, and a decreased quality of life. This study aimed to evaluate the effect of adding buprenorphine and magnesium to bupivacaine for superficial parasternal intercostal plane blocks (SPIB) on pain and opioid consumption in the first 24 hours after coronary artery bypass grafting (CABG).

Methods

Patients undergoing CABG were divided into the following four groups: saline SPIB, SPIB with bupivacaine (BPVC), SPIB with bupivacaine and buprenorphine (BPVC+BPRN), and SPIB with bupivacaine, buprenorphine, and magnesium (BPVC+BPRN+MG). The primary outcomes were pain scores and opioid consumption after SPIB; the secondary outcomes were post-operative nausea and vomiting, time to extubation, and length of stay (LOS) in the intensive care unit and hospital.

Results

One hundred thirty-four eligible patients undergoing CABG were randomized to either the saline (n=27), BPVC (n=20), BPVC+BPRN (n=24), or BPVC+BPRN+MG (n=29) group. All of the intervention groups combined (BPVC, BPVC+BPRN, and BPVC+BPRN+MG) had decreased pain scores and decreased opioid consumption when compared to the saline group; although not statistically significant, visual analog scale (VAS) scores trended downward at most time points with BPVC versus saline, BPVC+BPRN versus BPVC, and BPVC+BPRN+MG versus BPVC+BPRN. There was no difference among the study groups in the incidence of post-operative nausea and/or vomiting (PONV), time to extubation, hospital LOS, and ICU LOS.

Conclusion

In this prospective, double-blind, placebo-controlled trial, we found that SPIB with local anesthetic is effective at reducing VAS scores and opioid consumption after CABG. Further study is needed to determine whether the addition of adjuvants can further improve pain control and opioid consumption.

## Introduction

Perioperative pain management continues to be a significant challenge in cardiac surgery after sternotomy. Inadequate postoperative analgesia results in increased morbidity, impaired physical function and delay in recovery, increased perioperative and chronic opioid consumption, increased cost of care, and a decreased quality of life [1]. Additionally, the incidence of postoperative chronic pain six months after surgery and two years after cardiac surgery has been reported to be 37% and 17%, respectively [2]. Preventive strategies to avoid the development of chronic pain after surgery include changing surgical technique, maintaining adequate pain control throughout the perioperative period (including using regional techniques), and preoperative intervention focused on psychosocial and cognitive risk factors [3].

Traditionally, opioids have been the mainstay for perioperative pain management in patients undergoing cardiac surgery. However, a range of undesired effects including excessive sedation, respiratory depression, pulmonary aspiration, nausea, vomiting, constipation, urinary retention, pruritis, and ileus have led to the institution of multimodal analgesic regimens that include non-opioid analgesics and regional analgesic techniques in the perioperative period [4]. Neuraxial techniques have also been used to manage pain, but, despite evidence of neuraxial anesthesia being beneficial during cardiac surgery, their use is controversial because of the need for systemic heparinization during cardiopulmonary bypass which may increase the risk for bleeding and neurologic injury [5-8].

Commonly used regional analgesic techniques for cardiac surgery to manage postoperative pain include pectoralis nerve blocks, serratus anterior plane blocks, erector spinae plane blocks, paravertebral blocks, transversus thoracic muscle plane blocks, and parasternal intercostal nerve blocks. These techniques have been shown to provide improved postoperative pain control and decrease opioid requirements while leading to fewer postoperative complications [9-13]. Superficial parasternal intercostal plane blocks (SPIB) have been shown to be effective in patients having sternal pain after cardiac surgery; these blocks target the anterior cutaneous branches of the intercostal nerves [14-16]. For this block, local anesthetic is typically administered at multiple levels near the sternum; the injections can be administered under direct visualization into the surgical field or using ultrasound guidance after chest closure [17].

While current research suggests regional analgesic techniques are effective for pain reduction after cardiac surgery, the ideal formulation of local anesthetic and adjuvant medications necessary to provide an effective, and long-lasting block is still undefined. Bupivacaine is a local anesthetic that inhibits sodium channels that propagate action potentials in axons, dendrites, and muscles; additionally, bupivacaine inhibits N-methyl-D-aspartate (NMDA) receptor-mediated synaptic transmission in the spinal cord [18,19]. Magnesium sulfate is an NMDA receptor antagonist and exerts its analgesic effects by at least two mechanisms - it inhibits the inflammatory response by reducing cytokine delivery and acts as a calcium antagonist by blocking NMDA receptors [20,21]. Buprenorphine is a partial opioid receptor agonist that has a higher potency, slower onset, and longer duration of action than other long-acting opioids. It acts as a partial mu opioid receptor agonist and a kappa opioid receptor antagonist [22]. Both magnesium and buprenorphine, individually, have been hypothesized to have potential synergistic effects when combined with local anesthetic; magnesium also affects NMDA receptors and buprenorphine potentiates the effect of local anesthetic by hyperpolarization of afferent sensory neurons [23]. Both buprenorphine and magnesium have been previously studied individually as adjuvants to local anesthetic in regional anesthesia, but, to date, no studies have examined the addition of both buprenorphine and magnesium to local anesthetic in SPIB following cardiac surgery.

The primary objective of this study was to determine if the addition of buprenorphine and magnesium to bupivacaine for SPIB would reduce pain scores and opioid consumption in the first 24 hours after sternotomy for coronary artery bypass grafting (CABG). The secondary outcomes evaluated include the incidence of post-operative nausea and/or vomiting (PONV), time to extubation, and length of stay (LOS) in the intensive care unit (ICU) and in the hospital.

According to our hypothesis, the addition of magnesium and buprenorphine to bupivacaine for SPIB would decrease pain scores and opioid consumption within the first 24 hours after CABG, decrease the incidence of PONV, decrease the time to extubation, and decrease the duration of both surgical intensive care unit (SICU) and hospital LOS, when compared to SPIB done with buprenorphine and bupivacaine, bupivacaine alone, and saline placebo.

## Materials and methods

This prospective, placebo-controlled, randomized, double-blind study was performed at St. Joseph Mercy Oakland Hospital in Pontiac, Michigan. Institutional review board approval was obtained from St. Joseph Mercy Oakland Hospital; the study was registered at clinicaltrials.gov (NCT05003765). The investigators enrolled 134 consecutive patients undergoing isolated CABG with one of three cardiothoracic surgeons between August 2021 and June 2022. Written informed consent was obtained from each patient in the pre-operative holding area prior to surgery.

Participants

Patients between 18 and 100 years of age were eligible. Men and women of all races and ethnic backgrounds undergoing isolated CABG under general anesthesia were enrolled.

Patients were excluded based on the following criteria: patient refusal, emergency surgery, redo sternotomy, left ventricular ejection fraction <40%, preexisting pulmonary disease (pulmonary fibrosis, severe chronic obstructive pulmonary disease) or neurologic dysfunction, allergy to local anesthetics, significant hepatic or renal disease, unexpected additional valvular or aortic procedures during the scheduled CABG operations, chronic opioid use, and continued intubation for greater than 6 hours after surgery.

Intraoperative management

All participating patients received general endotracheal anesthesia with transesophageal echocardiography during their operations. Intraoperative opioid administration was left to the discretion of the cardiac anesthesiologist. SPIB (ribs 2-6 bilaterally) were performed by a single cardiothoracic surgery physician assistant with saline (control), 0.25% bupivacaine, 0.25% bupivacaine and 300 mcg buprenorphine, or 0.25% bupivacaine, 300 mcg buprenorphine, and 200 mg magnesium sulfate.

**Figure 1:**
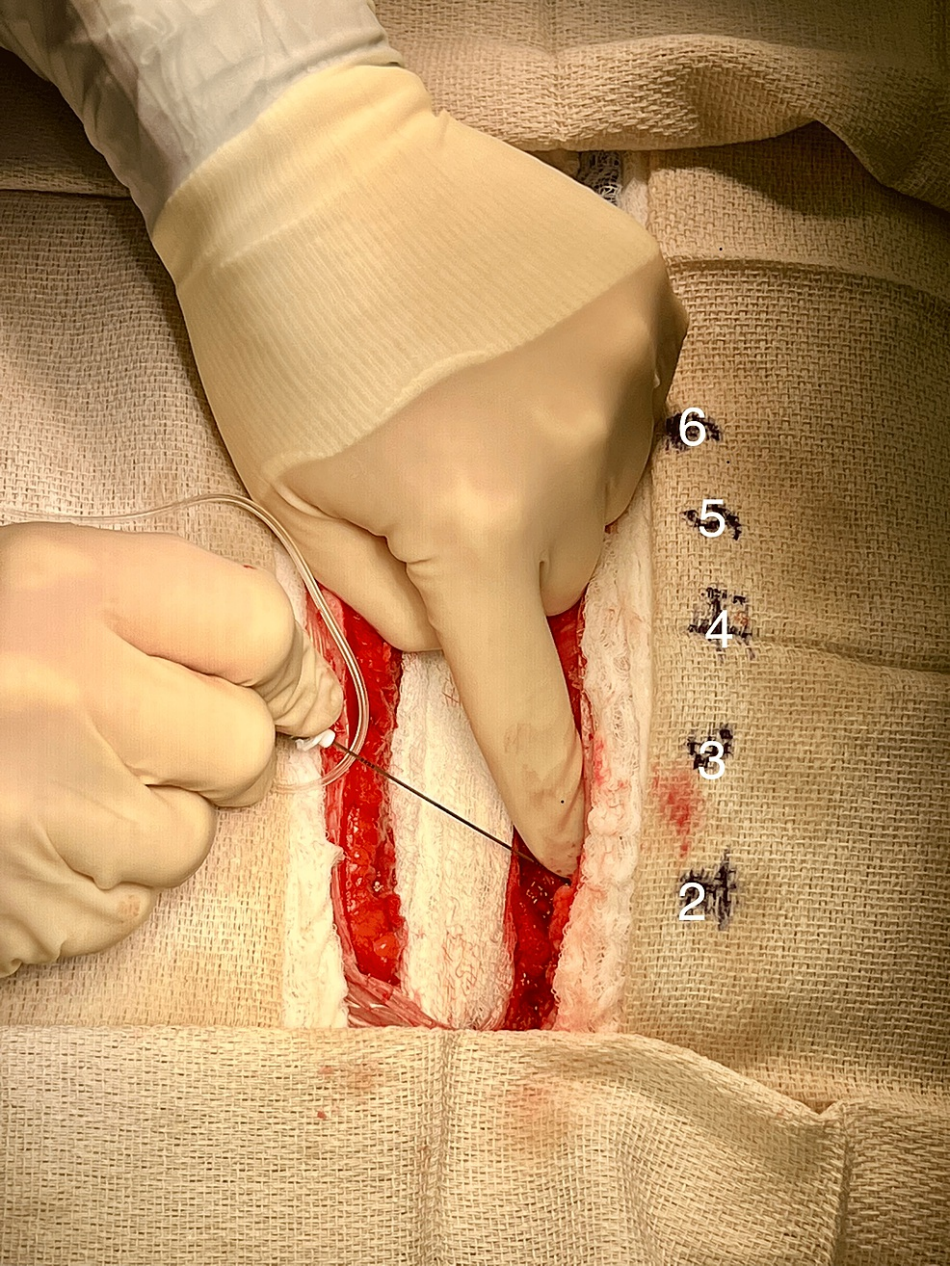
Injection of local anesthetic below ribs 2-6 during SPIB prior to chest closure. SPIB: superficial parasternal intercostal plane blocks

Patients were randomly assigned to one of four groups using a computer-generated random number table. Participants, the cardiothoracic surgery team, the anesthesiology staff, and the nurses caring for the patients were blinded to group allocation. Patients in group 1 (saline {control}) received SPIB using 40 mL of sterile saline. Below each rib (2-6) deep to the pectoralis major muscle and superficial to the intercostal muscles 4 mL of the solution was injected. Acceptable local anesthetic injection at each level was verified using direct visualization prior to sternal closure (Figure 1). Patients in group 2 (BPVC) received 40 mL of 0.25% bupivacaine. Patients in group 3 (BPVC+BPRN) received 40 mL of 0.25% bupivacaine and 300 mcg of buprenorphine. Patients in group 4 (BPVC+BPRN+MG) received 40 mL of 0.25% bupivacaine, 300 mcg of buprenorphine, and 200 mg of magnesium sulfate. All SPIB were performed under direct visualization prior to chest closure in the operating room.

Data collection

Patients were extubated in the ICU after meeting standard extubation criteria including normothermia, adequate respiratory function, alertness, hemostasis, and hemodynamic stability. Post-operative pain was documented by ICU nursing staff at extubation and every 6 hours using a visual analog scale (VAS) over the ensuing 24 hours. Post-operative administration of opioids and other pain medications was determined by the critical care and cardiothoracic surgery teams. To facilitate comparison, total opioid consumption for all patients in this study was converted to oral morphine equivalents (OME). The following conversion equivalents were used: 5 mg morphine (PO)=5 mg hydrocodone (PO)=3.3 mg oxycodone (PO)=1.25 mg hydromorphone (PO)=0.25 mg hydromorphone (IV)=0.02 mg fentanyl (IV)=1.67 mg morphine (IV/IM/SC)=50 mg tramadol=50 mg meperidine (PO)=12.5 mg meperidine (IV/IM/SC)=33.33 mg codeine (PO)=20 mg codeine (IV/IM/SC) based on the recommendations of http://www.globalrph.com/narcotic.cgi.

Statistical analysis

The primary outcomes of the investigation were total opioid consumption for (OME {mg}) 24 hours post-surgery and pain scores at time of extubation and 6, 12, 18, and 24 hours post-operation. Secondary outcomes included time from end of surgery to extubation, hospital LOS, ICU LOS, and incidence (percentage of the patients) of PONV requiring treatment during the first 24 hours following surgery. All outcomes were analyzed as continuous variables except for the incidence of PONV, which was treated as a categorical variable.

Demographic data were analyzed using t-tests, χ^2^ tests, and Fisher exact tests, as appropriate. For the continuous data, the statistical differences between the four groups of the study, saline (n=27), BPVC (n=20), BPVC+BPRN (n=24), and BPVC+BPRN+MG (n=29), were determined using first by a one-way analysis of variance (ANOVA) and then by two-by-two comparison using the post-hoc Fischer Least Significant Difference (LSD) test. For the categorical data, the statistical difference was determined using Pearson χ^2^, followed by Yates continuity correction and the Fisher exact probability test. A p-value of less than 0.05 was considered to be statistically significant. A p-value of 0.05 <p <0.1 (significant one-tailed test) can indicate a trend that could become significant with stronger power in the study. Data are reported as number (percentage of patients) or mean±SEM (mean±standard error of mean) or mean±standard deviation (mean±SD). All the data were analyzed using SPSS version 24 (Armonk, NY: IBM Corporation).

## Results

**Figure 2:**
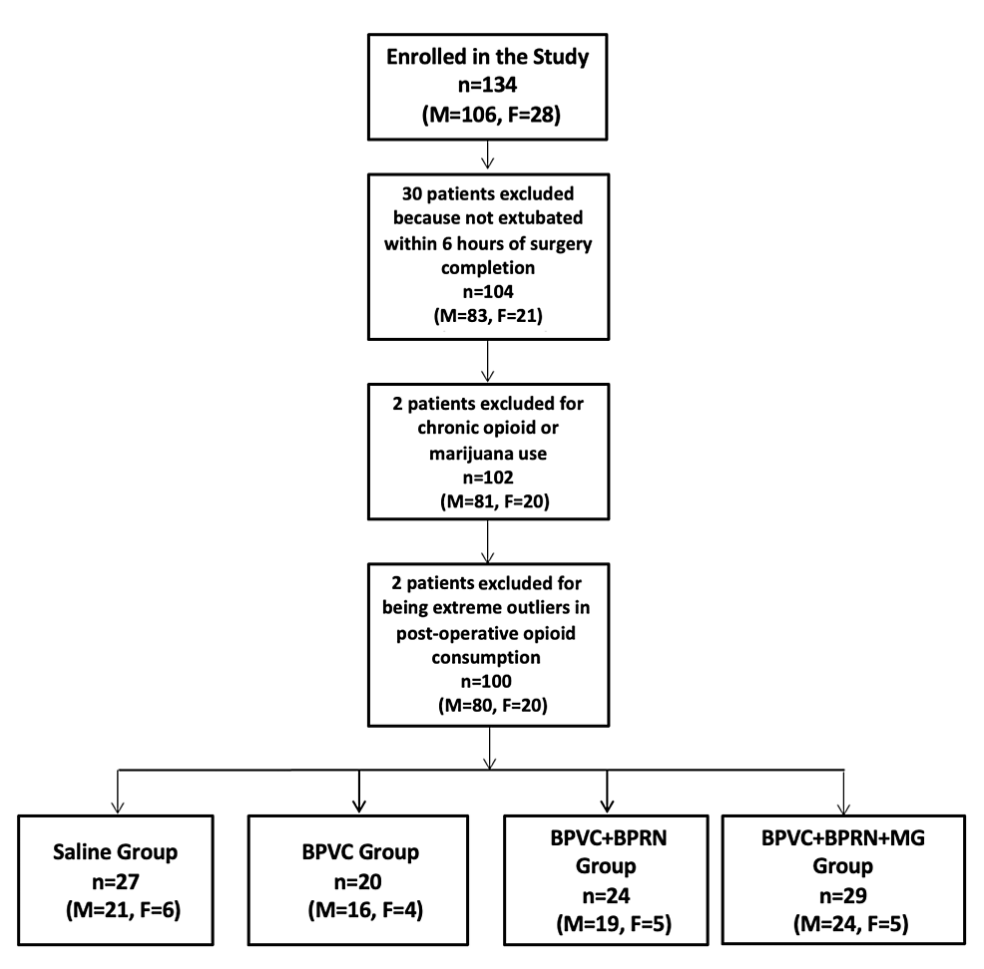
Consolidated Standards of Reporting Trials (CONSORT) diagram. BPVC: bupivacaine; BPRN: buprenorphine; MG: magnesium

Figure 2 displays the Consolidated Standards of Reporting Trials (CONSORT) diagram for this study. A total of 134 patients were enrolled in the study. Data for 34 patients were excluded, resulting in a total of 100 patients divided into the four study groups: saline (n=27), BPVC (n=20), BPVC+BPRN (n=24), and BPVC+BPRN+MG (n=29). Thirty patients were excluded for not being extubated within 6 hours of completion of surgery; these patients were excluded so as not to miss pain score data points. One patient was excluded due to chronic opioid use and one patient was excluded due to chronic marijuana use. Two patients were also excluded because they were extreme outliers in terms of postoperative opioid consumption (see CONSORT diagram in Figure 2).

Table 1 summarizes patient demographics and perioperative data including age, gender, race, weight, and BMI listed separately for all four groups of the study. Table 2 lists the results of comparing pain scores between the saline (n=27) group and the combination of the other three SPIB groups (n=73) for the five time points (extubation time: 6, 12, 18, and 24 hours) and total post-operative 24-hour opioid consumption (OME) (mg). Table 3 lists pain scores at extubation and at 6, 12, 18, and 24 hours after the surgery for all four groups of the study separately. Table 4 summarizes opioid consumption including total intra-operative opioid OME (mg), total post-operative 24-hour opioid OME (mg), and total opioid consumption OME (mg) for all four groups. Table 5 lists the summary of results for the duration of surgery (hours), time to extubation after surgery (hours), length of hospital stay (days), and length of ICU stay (days) variables for all fur groups of the study. Table 5 also lists the number of patients with PONV in the first 24 hours after surgery for all four groups of the study.

**Table 1:** Patient demographics for all four groups of the study. Values are mean±SD or number (percentage of patients). P<0.05 is considered statistically significant. Significant differences due to the power considerations only for all (male+female) and males are shown. BPVC: bupivacaine; BPRN: buprenorphine; MG: magnesium

Variables		Saline (n=27)	BPVC (n=20)	BPVC+BPRN (n=24)	BPVC+BPRN+MG (n=29)
Gender	Male	21 (26)	16 (20)	19 (24)	24 (30)
	Female	6 (30)	4 (20)	5 (25)	5 (25)
Race	African American	1 (4)	0 (0)	0 (0)	3 (10)
	Asian American	0 (0)	1 (5)	0 (0)	0 (0)
	Caucasian	26 (96)	19 (95)	24 (100)	25 (86)
	Latino American	0 (0)	0 (0)	0 (0)	1 (4)
Age (years)	All	66.8±7.1	66.2±10.4	65.5±7.7	68.1±8.4
	Male	68.2±7.2	64.4±10.8	65.6±6.9	68.5±8.5
	Female	61.8±4.4	73.3±4.2	65.2±11.5	65.8±8.2
Weight (kg)	All	93.5±15.8	99.8±35.1	93.4±16.0	90.6±15.1
	Male	96.0±15.0	103.6±38.3	94.2±14.6	92.1±15.7
	Female	84.8±16.7	85.1±9.6	90.6±22.3	83.7±10.9
BMI (kg/m^2^)	All	31.5±5.8	32.6±9.2	30.8±5.4	30.0±5.0
	Male	31.3±4.5	32.7±10.4	30.0±4.0	29.3±4.7
	Female	32.5±9.8	32.1±1.1	33.8±8.9	33.1±5.8

**Table 2:** Pain scores and total post-operative 24-hour opioid oral morphine equivalents. *Significantly different from saline (no-block) group (p<0.05). **A trend 0.05 <p <0.1. Values are mean±SD or number (percentage of patients). BPVC: bupivacaine; BPRN: buprenorphine; MG: magnesium; SPIB: superficial parasternal intercostal plane blocks

Variables		Saline SPIB (n=27)	BPVC, BPVC+BPRN, BPVC+BPRN+MG SPIB groups combined (n=73)	p-Value
Pain score at extubation	All	5.4±2.9	3.6±2.9*	0.011
	Male	5.3±3.0	3.5±2.7*	0.014
	Female	6.0±2.7	4.3±3.6	0.399
Pain score at 6 hours	All	5.5±3.0	3.8±2.6*	0.007
	Male	5.6±3.2	3.6±2.6*	0.006
	Female	5.3±2.6	4.9±2.9	0.729
Pain score at 12 hours	All	5.4±2.6	3.9±2.6*	0.010
	Male	5.3±2.5	3.7±2.6*	0.014
	Female	5.8±3.1	4.8±2.8	0.468
Pain score at 18 hours	All	6.0±1.7	4.1±2.7*	0.001
	Male	6.1±1.8	3.7±2.6*	0.001
	Female	6.0±1.4	5.4±2.7	0.659
Pain score at 24 hours	All	5.0±2.6	3.7±2.6*	0.030
	Male	4.7±2.5	3.6±2.3**	0.067
	Female	6.0±3.0	4.2±3.5	0.296
Total post-operative 24-hour opioid oral morphine equivalents (mg)	All	91.0±40.9	48.2±32.6*	0.000
	Male	92.1±39.3	50.6±35.1*	0.000
	Female	87.1±50.1	37.9±16.3*	0.003

**Table 3:** Pain scores for all four groups of the study. *Significantly different from saline group. **Significantly different from BPVC group. ***Significantly different from BPVC+BPRN group. Values are mean±SD or number (percentage of patients). P-value<0.05 is considered statistically significant. Significant differences due to the statistical power considerations only for all (male+female) and males are shown. BPVC: bupivacaine; BPRN: buprenorphine; MG: magnesium

Variables		Saline (n=27)	BPVC (n=20)	BPVC+BPRN (n=24)	BPVC+BPRN+MG (n=29)
Pain score at Extubation	All	5.4±2.9	5.0±2.7	3.7±3.0*	2.6±2.6^*,**^
	Male	5.2±3.0	3.8±2.2	3.7±2.3	2.7±3.1^*,**^
	Female	6.0±2.7	5.7±1.2	3.8±5.2	2.8±1.6
Pain score at 6 hours	All	5.5±3.0	4.3±3.5	4.1±2.8	3.3±1.7^*^
	Male	5.6±3.2	4.1±3.5	3.9±2.6	3.0±1.5^*^
	Female	5.3±2.6	5.3±3.6	4.8±4.0	4.6±1.8
Pain score at 12 hours	All	5.4±2.6	4.8±2.8	3.7±2.7*	3.4±2.4^*^
	Male	5.3±2.5	4.2±2.9	3.6±2.7*	3.3±2.3^*^
	Female	5.8±3.1	7.0±0.8	3.8±3.0	3.8±3.0
Pain score at 18 hours	All	6.0±1.7	4.1±3.0*	4.9±2.6	3.4±2.5^*,***^
	Male	6.1±1.8	4.0±3.1*	4.2±2.4*	3.2±2.5^*^
	Female	6.0±1.4	4.3±3.3	7.6±1.1	4.2±2.2
Pain score at 24 hours	All	5.0±2.6	4.5±2.6	3.5±2.4*	3.4±2.7^*^
	Male	4.7±2.5	4.4±2.4	3.3±2.1	3.3±2.4
	Female	6.0±3.0	4.8±3.6	4.2±3.6	3.8±4.1

**Table 4:** Opioid consumption for all four groups of the study. *Significantly different from saline group. **Significantly different from BPVC group. ***Significantly different from BPVC+BPRN group. Values are mean±SD or number (percentage of patients). P-value <0.05 is considered statistically significant. Significant differences due to the statistical power considerations only for all (male+female) and males are shown. BPVC: bupivacaine; BPRN: buprenorphine; MG: magnesium

Variables		Saline (n=27)	BPVC (n=20)	BPVC+BPRN (n=24)	BPVC+BPRN+MG (n=29)
Total intra-operative opioid - oral morphine equivalents (mg)	All	307.4±75.4	275.0±30.9	284.9±49.6	269.0±62.6^*^
	Male	321.3±67.4	269.0±47.7	290.1±46.9	280.9±62.3
	Female	291.7±67.9	262.5±12.5	265.0±60.2	212.5±56.6
Total post-operative 24-hour opioid - oral morphine equivalents (mg)	All	91.0±40.9	41.1±9.9^*^	60.9±32.7^*^	42.6±39.8^*^
	Male	93.7±39.5	38.6±12.6^*^	67.6±32.4^*,**^	47.2±47.5^*,***^
	Female	87.1±50.1	48.4±6.2	35.2±19.2	32.3±17.9
Total opioid consumption - oral morphine equivalents (mg)	All	398.4±93.4	316.1±30.1^*^	345.8±63.8^*^	311.5±87.1^*^
	Male	415.0±91.2	307.7±46.0^*^	357.8±61.3	328.1±92.9^*^
	Female	378.8±54.1	310.9±17.7	300.2±56.9	244.8±65.5

**Table 5:** Surgery duration, time to extubation, length of stay, and PONV. *Significantly different from saline group. **Significantly different from BPVC group. ***Significantly different from BPVC+BPRN group. Values are mean±SD or number (percentage of patients). P-value <0.05 is considered statistically significant. Significant differences due to the statistical power considerations only for all (male+female) and males are shown. BPVC: bupivacaine; BPRN: buprenorphine; MG: magnesium; PONV: post-operative nausea and/or vomiting

Variables		Saline (n=27)	BPVC (n=20)	BPVC+BPRN (n=24)	BPVC+BPRN+MG (n=29)
Duration of surgery (hours)	All	6.1±0.9	5.9±0.8	6.1±0.9	6.0±0.8
	Male	6.1±0.9	6.0±0.8	6.2±0.7	6.1±0.8
	Female	6.0±0.7	5.9±0.7	5.7±1.5	5.7±1.0
Time to extubation after surgery (hours)	All	5.1±1.7	4.7±1.3	5.2±1.4	4.8±1.7
	Male	4.9±1.7	4.7±1.4	5.0±1.5	4.6±1.5
	Female	5.8±2.0	4.6±0.8	5.7±0.9	5.7±2.5
Length of Hospital stay (days)	All	5.9±1.6	6.5±3.3	6.0±2.2	5.8±3.1
	Male	5.6±1.6	6.4±3.7	6.2±2.3	5.4±1.7
	Female	6.8±1.4	6.8±0.5	5.6±1.5	7.6±6.5
Length of ICU stay (days)	All	2.9±1.3	2.0±0.0*	3.4±1.6**	2.3±0.9***
	Male	2.9±1.4	2.0±0.0*	3.3±1.8**	2.3±0.8***
	Female	2.8±1.0	2.0±0.0	3.8±0.4	2.6±1.3
Number of patients with PONV in first 24 hours	All	Yes=13 (48)	Yes=10 (50)	Yes=7 (29)	Yes=13 (45)
		No=14 (52)	No=10 (40)	No=17 (71)	No=16 (55)
	Male	Yes=10 (48)	Yes=7 (44)	Yes=4 (21)	Yes=10 (42)
		No=11 (52)	No=9 (56)	No=15 (79)	No=14 (58)
	Female	Yes=3 (50)	Yes=3 (75)	Yes=3 (60)	Yes=3 (60)
		No=3 (50)	No=1 (25)	No=2 (40)	No=2 (40)

**Figure 3:**
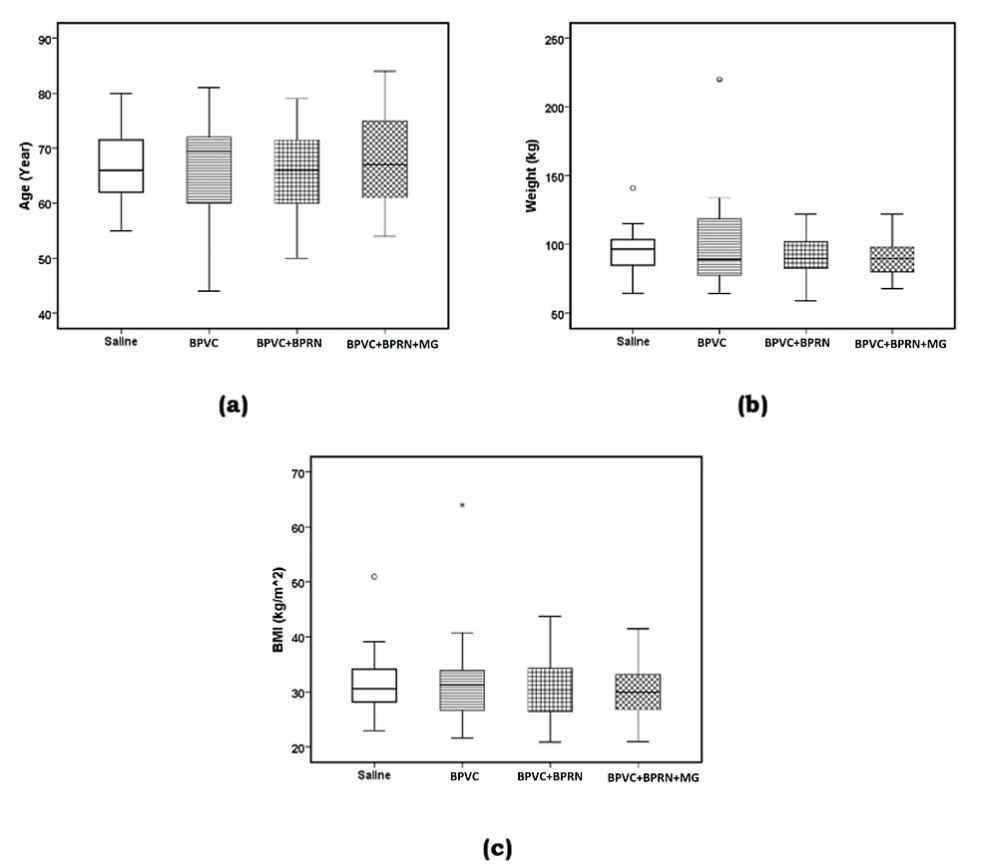
Patient characteristics for all four groups of the study. Boxplots of (a) age (year), (b) weight (kg), and (c) BMI (body mass index) (kg/m^2^). The solid line in the middle of the box represents the median. The box represents the middle 50%, and the whiskers represent the top and bottom 25%. Mild outliers are represented by circles and extreme outliers by stars. There was no significant difference between the mean values of the four groups of all three variables. BPVC: bupivacaine; BPRN: buprenorphine; MG: magnesium

**Figure 4:**
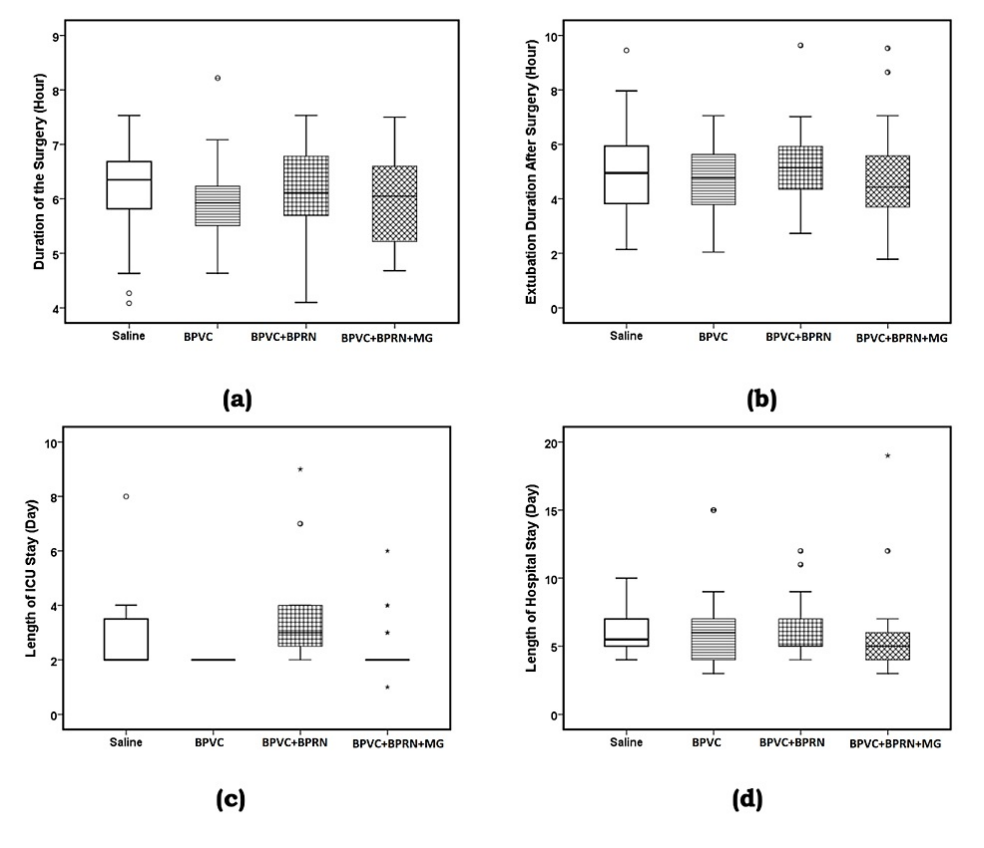
Boxplots of (a) duration of the surgery (hours), (b) time to extubation after surgery (hours), (c) length of ICU stay (days), and (d) length of hospital stay (days). The solid line in the middle of the box represents the median. The box represents the middle 50%, and the whiskers represent the top and bottom 25%. Mild outliers are represented by circles and extreme outliers by stars. BPVC: bupivacaine; BPRN: buprenorphine; MG: magnesium

There was no significant difference between the four groups of the study with respect to gender (Pearson χ^2^= 0.23, p=0.9726) and race (Pearson χ^2^=11.43, p=0.2474) (Table 1). There was no significant difference between the four groups of the study in the ANOVA results or the six possible post-hoc LSD comparisons with respect to age (F {3, 96}=0.456, p=0.714, Table 1, Figure 3, panel a); weight (F {3, 96}=0.784, p=0.506, Table 1, Figure 3, panel b); BMI (F {3, 94}=0.712, p=0.547, Table 1, Figure 3, panel c); duration of surgery (F {3, 96}=0.234, p=0.872, Table 5, Figure 4, panel a) and time to extubation after surgery (F {3, 96}=0.506, p=0.679, Table 5, Figure 4, panel b).

**Figure 5:**
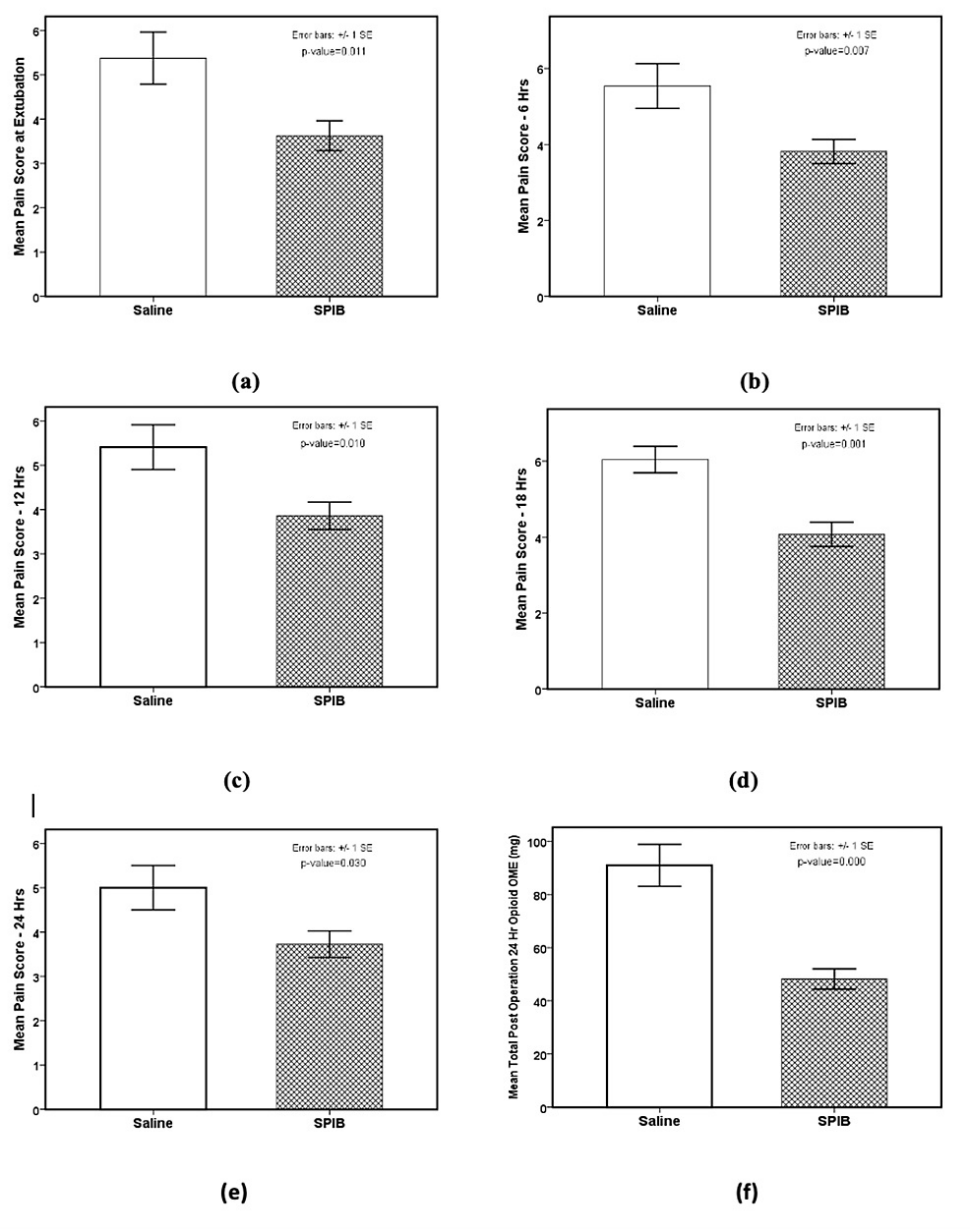
Comparison of saline (n=27) group and the three (BPVC, BPVC+BPRN, and BPVC+BPRN+MG) SPIB groups with bupivacaine combined (n=73). The pain scores and the total post-operative 24-hour opioid OME (mg) are significantly lower for the three SPIB groups with bupivacaine combined in comparison to the saline group. The p-values are as the following: (a) 0.011, (b) 0.007, (c) 0.010, (d) 0.001, (e) 0.030, and (f) 0.000. BPVC: bupivacaine; BPRN: buprenorphine; MG: magnesium; SPIB: superficial parasternal intercostal plane blocks

For demonstrating the efficacy of the SPIB, the pain scores at extubation, 6, 12, 18, and 24 hours after surgery and total post-operative 24-hour opioid consumption (OME) (mg) were compared between the saline (n=27) group and the three other block groups combined (BPVC, BPVC+BPRN, and BPVC+BPRN+MG) (n=73). ANOVA analysis results were the following: pain score at extubation (F {1, 94}=6.708, p=0.011, w=0.237 {small to medium effect size}, Table 2, Figure 5, panel a); pain score at 6 hours (F {1, 94}=7.493, p=0.007, w=0.252 {small to medium effect size}, Table 2, Figure 5, panel b); pain score at 12 hours (F {1, 97}=6.827, p=0.010, w=0.236 {small to medium effect size}, Table 2, Figure 5, panel c); pain score at 18 hours (F {1, 95}=11.532, p=0.001, w=0.313 {medium effect size}, Table 2, Figure 5, panel d); pain score at 24 hours (F {1, 98}=4.834, p=0.030, w=0.192 {small effect size}, Table 2, Figure 5, panel e) and total post-operative 24-hour opioid OME (mg) (F {1, 98}=29.374, p=0.000, w=0.470 {large effect size}, Table 2, Figure 5, panel f).

**Figure 6:**
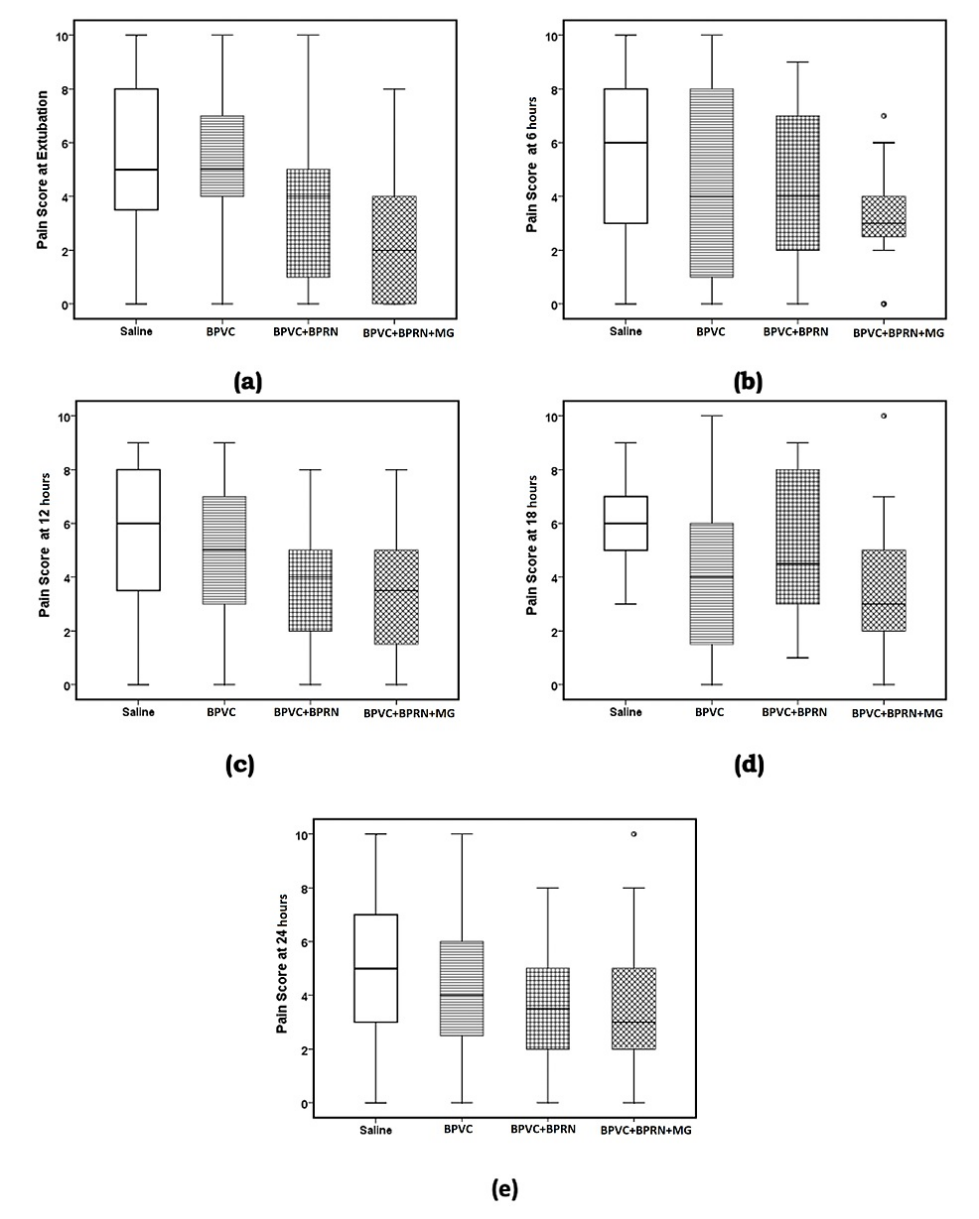
Boxplots (a) pain score at extubation, (b) pain score at 6 hours, (c) pain score at 12 hours, (d) pain score at 18 hours, and (e) pain score at 24 hours. The solid line in the middle of the box represents the median. The box represents the middle 50%, and the whiskers represent the top and bottom 25%. Mild outliers are represented by circles and extreme outliers by stars. BPVC: bupivacaine; BPRN: buprenorphine; MG: magnesium

**Figure 7:**
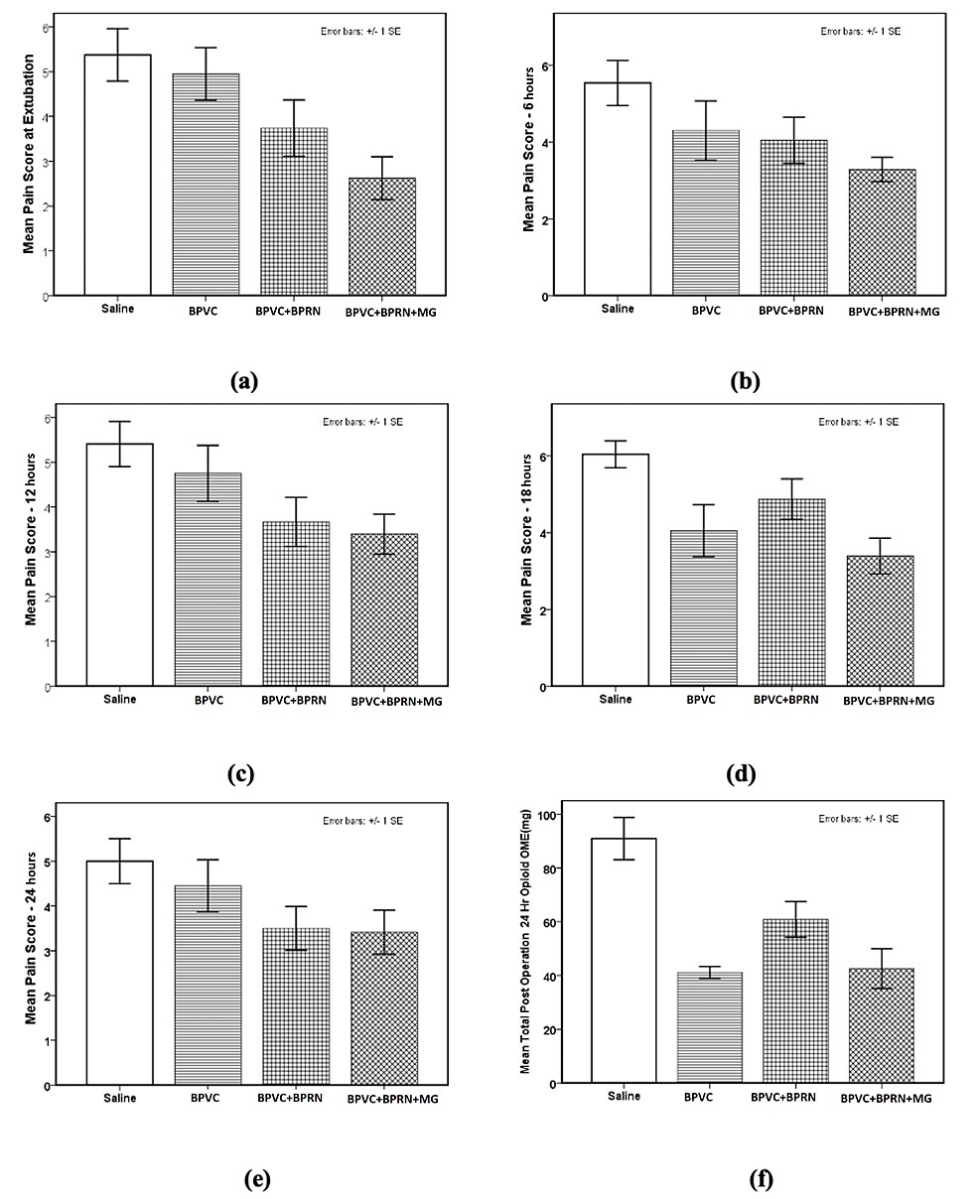
Bar charts for the pain scores at (a) extubation, (b) 6 hours, (c) 12 hours, (d) 18 hours, (e) 24 hours, and (f) the total post-operative 24-hour opioid consumption (OME) (mg) for all four groups. At extubation (a) pain scores for the BPVC+BPRN (p=0.046) and BPVC+BPRN+Mg (p=0.001) groups were significantly lower than the saline group. Pain scores for the BPVC+BPRN+Mg (p=0.005) group were significantly lower than the BPVC group. At 6 hours (b) pain scores for the BPVC+BPRN+Mg (p=0.003) group were significantly lower than saline group. At 12 hours (c) pain scores for the BPVC+BPRN (p=0.019) and BPVC+BPRN+Mg (p=0.005) groups were significantly lower than the saline group. At 18 hours (d) pain scores for the BPVC (p=0.008) and BPVC+BPRN+Mg (p=0.000) groups were significantly lower than the saline group. Pain scores for the BPVC+BPRN+Mg (p=0.003) group were also significantly lower than BPVC+BPRN group. At 24 hours (e) pain scores for the BPVC+BPRN (p=0.040) and BPVC+BPRN+Mg (p=0.023) groups were significantly lower than the saline group. The total post-operative 24-hour opioid consumption (f) for the BPVC (p=0.000), BPVC+BPRN (p=0.002), and BPVC+BPRN+Mg (p=0.000) groups were significantly lower when compared to the saline group. Error bars are ±1 x SEM (standard error mean). BPVC: bupivacaine; BPRN: buprenorphine; MG: magnesium

ANOVA analysis for the pain score variables for the four groups was the following: pain score at extubation (F {3, 92}=5.190, p=0.002, w=0.340 {medium effect size}, Table 3, Figure 6, panel a and Figure 7, panel a); pain score at 6 hours (F {3, 92}=3.100, p=0.031, w=0.248 {small to medium effect size}, Table 3, Figure 6, panel b and Figure 7, panel b); pain score at 12 hours (F {3, 95}=3.431, p=0.020, w=0.262 {small to medium effect size}, Table 3, Figure 6, panel c and Figure 7, panel c); pain score at 18 hours (F {3, 93}=5.510, p=0.002, w=0.350 {medium effect size}, Table 3, Figure 6, panel d and Figure 7, panel d) and pain score at 24 hours (F {3, 96}=2.348, p=0.077, w=0.197 {small effect size}, Table 3, Figure 6, panel e). Post-hoc LSD test showed that mean value of the pain scores for the BPVC+BPRN+MG group was significantly reduced in comparison to the saline group for all five-time points - pain score at extubation (p=0.001, Table 3, Figure 6, panel a and Figure 7, panel a); pain score at 6 hours (p=0.003, Table 3, Figure 6, panel b and Figure 7, panel b); pain score at 12 hours (p=0.005, Table 3, Figure 6, panel c and Figure 7, panel c); pain score at 18 hours (p=0.000, Table 2, Figure 6, panel d and Figure 7, panel d); and pain score at 24 hours (p=0.023, Table 3, Figure 6, panel e and Figure 7, panel e). Similarly, post-hoc LSD test showed that mean value of pain scores for the BPVC group was significantly reduced in comparison to the saline group at 18 hours (p=0.008, Table 3, Figure 6, panel d and Figure 7, panel d). Post-hoc LSD test also showed that the mean value of the pain score for the BPVC+BPRN group was significantly reduced in comparison to the saline group at extubation (p=0.046, Table 3, Figure 6, panel a and Figure 7, panel a), 12 hours (p=0.019, Table 2, Figure 5, panel c and Figure 6, panel c), and 24 hours (p=0.040, Table 3, Figure 6, panel e and Figure 7, panel e). Additionally, post-hoc LSD test showed that the mean value of the pain score for the BPVC+BPRN+MG group was significantly reduced in comparison to the BPVC+BPRN group at 18 hours (p=0.033, Table 3, Figure 6, panel d and Figure 7, panel d).

ANOVA analysis for the LOS in the ICU and hospital showed the following: length of ICU stay (days) (F {3, 96}=6.673, p=0.000, w=0.381 {medium effect size}, Table 5, Figure 4, panel c) and length of hospital stay (days) (F {3, 94}=0.320, p=0.811, Table 5, Figure 4, panel d). Post-hoc LSD test showed that the mean value of length of ICU stay for the BPVC group was significantly lower than the saline (p=0.015) and BPVC+BPRN (p=0.000) groups. Similarly, post-hoc LSD test showed that mean value of the length of ICU stay for the BPVC+BPRN+MG group was significantly lower than the mean value for the BPVC+BPRN group (p=0.001) (Table 5 and Figure 4, panel c).

**Figure 8:**
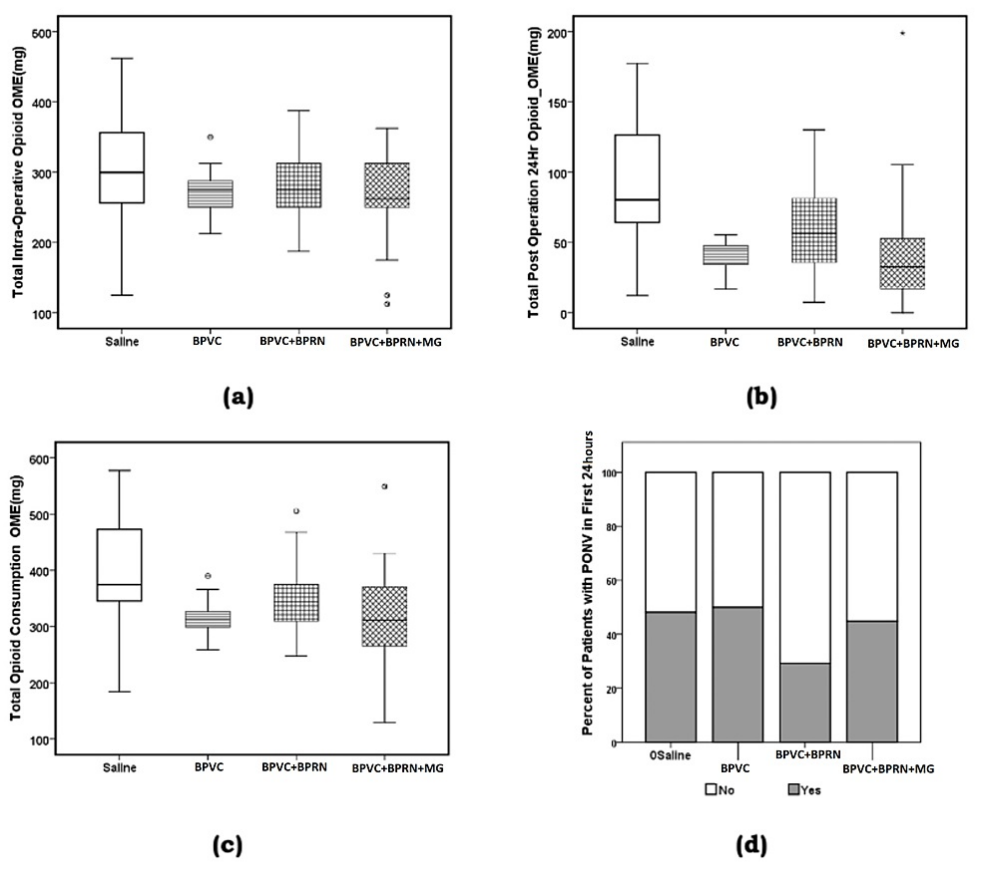
Boxplots (a) total intra-operative opioid oral morphine equivalent (mg), (b) total post-operative 24-hour opioid oral morphine equivalent (mg), (c) total opioid consumption oral morphine equivalent (mg), and (d) percent of the patients with PONV in the first 24-hours after surgery for the four groups. The solid line in the middle of the box represents the median. The box represents the middle 50%, and the whiskers represent the top, and bottom 25%. Mild outliers are represented by circles and extreme outliers by stars. BPVC: bupivacaine; BPRN: buprenorphine; MG: magnesium; PONV: post-operative nausea and/or vomiting

ANOVA analysis results for the opioid consumption variables were as the following: total intra-operative opioid OME (mg) (F {3, 96}=2.213, p=0.092, w=0.187 {small effect size} Table 4, Figure 8, panel a); total post-operation 24-hour opioid OME (mg) (F {3, 96}=11.689, p=0.000, w=0.493 {large effect size}, Table 4, Figure 7, panel f and Figure 8, panel b); total opioid consumption OME (mg) (F {3, 96}=7.328, p=0.000, w=0.399 {medium to large effect size}, Table 4, Figure 8, panel c). Post-hoc LSD test showed that mean value of total intra-operative opioid OME (mg) was significantly reduced for the BPVC+BPRN+MG (p=0.016) group in comparison to saline group. Similarly, post-hoc LSD test showed that mean value of the total post-operative 24-hour opioid OME (mg) was significantly reduced for the BPVC (p=0.000), BPVC+BPRN (p=0.015), and BPVC+BPRN+MG (p=0.000) groups in comparison to the saline group. Finally, post-hoc LSD test showed that mean value of the total opioid consumption OME (mg) was significantly reduced for the BPVC (p=0.000), BPVC+BPRN (p=0.002), and BPVC+BPRN+MG (p=0.000) groups in comparison to the saline group.

There was also no significant association between the type of treatment patients received (saline, BPVC, BPVC+BPRN, and BPVC+BPRN+MG) and the incidence of PONV, that is, the percentage of the patients who experienced PONV within the first 24 hours after surgery (Pearson's χ^2^=2.61, p=0.456; Fisher exact probability test, two-tailed p=0.467, Table 5, Figure 8, panel d).

## Discussion

This prospective, randomized, double-blinded, placebo-controlled study was performed to determine whether adding buprenorphine and magnesium to bupivacaine for SPIB would improve analgesia for patients after CABG versus buprenorphine and bupivacaine, bupivacaine alone, or placebo. The study showed that, when compared to placebo, BPVC+BPRN+MG SPIB reduced post-surgery VAS scores at extubation, 6 hours, 12 hours, 18 hours, and 24 hours (Table 3). Although not statistically significant, mean VAS scores trended downward at almost all time points (except 18 hours) with the BPVC SPIB compared to placebo, with BPVC+BPRN SPIB compared to BPVC SPIB alone, and with BPVC+BPRN+MG SPIB compared to BPVC+BPRN SPIB (Figure 5). Mean post-operative 24-hour opioid consumption was reduced in all groups when compared to placebo, but addition of buprenorphine or buprenorphine and magnesium did not result in a significant decrease in opioid consumption when compared to bupivacaine alone (Table 4). There was no difference among the study groups in the incidence of PONV, time to extubation, hospital LOS, and ICU LOS. Overall, when comparing all groups that received SPIB with bupivacaine (BPVC, BPVC+BPRN, BPVC+BPRN+MG) combined versus placebo, patients receiving SPIB with bupivacaine showed statistically significant reductions in pain score at all time points as well as a reduction in post-operative opioid consumption (Figure 4).

Cardiac surgeons and intensivists managing post-operative cardiac surgery patients are tasked with managing acute pain in the post-operative period with the knowledge that nearly 10% of cardiac surgery patients will continue to use opioids 90 days after surgery [24]. These clinicians have long understood the association between opioid-focused analgesic regimens and postoperative complications [4]. In a recent study of opioid-related adverse events in Medicare patients undergoing cardiac surgery, Allen et al. reported a documented opioid-related adverse event rate of 0.7%, but the group proceeded to reveal that the potential opioid-related adverse event rate may have been as high as 32.4%. Further, the authors found that opioid-related adverse events increased LOS, decreased reimbursement, and increased hospital expenses [25].

In an effort to reduce opioid-related complications, current analgesic protocols following cardiac surgery frequently include non-opioid medications and regional analgesic techniques. These multimodal approaches to pain management are intended to act on multiple sites in the pain pathway [26].

Regional analgesia for management of sternal incisional pain in cardiac surgery, and specifically SPIB, has been shown to be an effective technique to improve pain scores, reduce opioid consumption, and improve patient satisfaction [14,15,27]. Chen et al. studied the effects of SPIB with ropivacaine compared to saline placebo in 41 patients in China; they reported a 20% reduction in sufentanil use, decreased post-operative pain scores, and improved patient satisfaction in the SPIB group [14]. Similarly, in another prospective randomized placebo-controlled trial involving 88 cardiac surgery patients, Barr et al. found that pain scores were approximately 50% lower and morphine consumption was approximately 50% less in the ropivacaine SPIB group [15]. Lee et al. studied 79 patients undergoing CABG; they compared SPIB with liposomal bupivacaine to placebo and found that overall pain levels were lower in the liposomal bupivacaine group, but they found no significant difference in opioid consumption post-operatively, ICU LOS, hospital LOS, and time to extubation [27].

The addition of magnesium and buprenorphine, individually as adjuvants to local anesthetic during regional anesthesia, has been shown to increase the duration of regional blocks, decrease pain scores, decrease opioid consumption, and decrease side effects from increased opioid consumption [28-30]. However, there are very few studies examining the addition of adjuvant medications to local anesthetic in regional anesthesia specifically for cardiac surgery. Kamel et al. compared the effect of magnesium when added to local anesthetic in parasternal blocks following valve replacement surgery. The authors found that the magnesium and bupivacaine group had lower VAS scores and fentanyl consumption during the first 48 hours, as well as shorter time to extubation when compared to the bupivacaine-alone parasternal block group and the intravenous paracetamol and ketorolac group [31]. While there is little data on adjuvants in regional blocks for cardiac surgery, the effects of adding multiple adjuvants to local anesthetics in regional anesthesia for cardiac surgery have not been studied. Supporting the safety of a multiple adjuvant medication approach, Williams et al. studied the effects of a bupivacaine/clonidine/buprenorphine/dexamethasone combination when injected around the sciatic nerve in rats; they found that the solution produced a reversible nerve block without causing damage to the nerve or long-term motor or sensory deficits [32].

Our study is the first prospective trial comparing multiple adjuvant medications with local anesthetic, a single adjuvant medication with local anesthetic, and local anesthetic alone to placebo in cardiac surgery. Similar to others who reported on the benefits of SPIB in cardiac surgery, we found a significant reduction in pain scores and post-operative opioid consumption when combining all local anesthetic SPIB groups and comparing to placebo. However, we were not able to consistently demonstrate a statistically significant reduction in pain scores and post-operative opioid consumption between groups with one or more adjuvant medications added to the local anesthetic. Because opioid orders were written by individual cardiothoracic surgeons as “pro re nata” nursing orders, it was difficult to standardize the dose and specific opioid given to patients for a given VAS score. Nevertheless, there was a trend towards lower VAS scores in patients receiving buprenorphine or buprenorphine and magnesium with bupivacaine in their SPIB. Additionally, there were no significant differences in extubation time, ICU LOS, and hospital LOS. The Society of Thoracic Surgeons (STS) defines early extubation after cardiac surgery as within 6 hours; this time frame is an STS quality marker. However, the relationship between extubating patients within the 6-hour time frame and improved outcomes including decreased ICU LOS and hospital LOS is controversial; some have reported that this 6-hour extubation window is not rooted in physiologic evidence [33]. It is more likely that dynamics affecting extubation time, ICU LOS, and hospital LOS are multifactorial, and improved management of pain may not be sufficient to dramatically improve these outcomes.

Our study is not without limitations. Our patient population consisted primarily of Caucasian males, even though all patients meeting inclusion criteria were consecutively enrolled during the study period. This limited demographic profile, although the groups were not statistically demographically different from each other, may limit the generalizability of the current study; an additional larger study may be required to confirm our findings. In addition, all SPIB were performed without ultrasound guidance, limiting the ability to confirm appropriate deposition of injectate into the correct plane. Instead, SPIB were performed by a single cardiothoracic surgery physician assistant under direct visualization using a landmark technique. We elected to perform SPIB without ultrasound to avoid potential sterility issues and to reduce the potential for postoperative sternal wound infection. Finally, given the relatively small study group, the study may not have been adequately powered to show statistical significance when comparing VAS scores between groups. Although the groups trended towards lower VAS scores as local anesthetic and additional adjuvant medications were added, larger group sizes may have revealed statistically significant differences.

In summary, this is the first prospective, randomized, double-blind, placebo-controlled study investigating the use of bupivacaine plus multiple adjuvant medications in SPIB following cardiac surgery. Although we noted that VAS scores and post-operative opioid consumption were reduced in the intervention groups versus the placebo group, we were not able to definitively show that adding buprenorphine or buprenorphine and magnesium to bupivacaine provided better relief than bupivacaine alone; we did, however, see a trend toward lower pain scores in the groups with buprenorphine and buprenorphine and magnesium. We can definitively report that SPIB is effective at reducing VAS scores and opioid consumption after cardiac surgery (when compared to placebo), but further large-scale study to confirm the local anesthetic formulation that will have the greatest impact on pain control, opioid use, and reduction in side effects is indicated.

## Conclusions

Controlling perioperative pain after sternotomy for cardiac surgery continues to be a significant challenge to clinicians. SPIB is a simple and safe regional analgesic technique that has been shown to be effective in patients having sternal pain after cardiac surgery.

In this prospective, double-blind, placebo-controlled trial, we found that SPIB was effective at reducing pain scores and opioid consumption when compared to placebo. We also saw a trend toward lower pain scores in intervention groups with buprenorphine and buprenorphine and magnesium included in the SPIB. Further large-scale study is needed to confirm whether the addition of these adjuvant medications to local anesthetic in SPIB can significantly enhance pain relief, reduce opioid consumption, and decrease side effects.
